# Knowledge on Diet among the Hypertensive Patients in a Tertiary Care Center Nepal: A Descriptive Cross-sectional Study

**DOI:** 10.31729/jnma.4815

**Published:** 2020-02-29

**Authors:** Nabina Maharjan, Narayani Maharjan, Rui Li

**Affiliations:** 1 Department of Healthcare Management, School of Health Sciences, Wuhan University, Wuhan, China; 2 Department of Clinical Laboratory Science, Zhongnan Hospital of Wuhan University, Wuhan, Hubei, China

**Keywords:** *diet*, *exercise*, *hypertension*, *knowledge.*

## Abstract

**Introduction:**

Hypertension is one of the leading causes of death and disability in both developed and developing countries. The prevalence of hypertension is increasing rapidly worldwide. The aim of the study was to determine the knowledge of diet among hypertensive patients.

**Methods:**

This descriptive cross-sectional study was conducted using a structured questionnaire among 169 hypertensive patients at Kathmandu diabetes and thyroid center from May 2017 to July 2017 after taking ethical clearance from Nepal Health Research Council, Nepal. A convenience sampling method was used. Data was collected and entry was done in Statistical Package for the Social Sciences version 16.0 point estimate at 95% confidence interval was calculated along with frequency and proportion for binary data.

**Results:**

Out of total 169 participants enrolled in this study, only 79 (46.7%) had good knowledge and 90 (53.3%) had poor knowledge regarding diet. The mean age of participants was 54.68±13.91 years.

**Conclusions:**

This study revealed that the knowledge about diet among hypertensive patients is poor and this study suggests the need for a proper educational intervention to improve awareness and to control hypertension effectively.

## INTRODUCTION

Hypertension is a major public health problem worldwide and one of the leading causes of cardiovascular diseases. It is a chronic condition that can be prevented and treated, but if left untreated, it leads to severe life-threatening complications such as the brain, heart, and renal disorders which mostly lead to disability.^1^

The growing incidence of hypertension is due to the aging population, sedentary lifestyle, urbanization, physical inactivity, obesity, excessive salt intake, alcohol consumption, and persistent stress exposure. Hypertension can be controlled by both non-pharmacological therapy and pharmacological treatment.^2^ Lifestyle intervention also has the ability to decrease the need or quantity of medications in hypertensive and stops developing high blood pressure in non-hypertensive.^3^

The main objective of this study was to determine the knowledge of diet among hypertensive patients.

## METHODS

It is a descriptive cross-sectional study conducted at Kathmandu Diabetes and Thyroid Center, Jawalakhel, Nepal from May 2017 to July 2017. The ethical approval was obtained from the ethical review board of Nepal Health Research Council (Ref.: 883) and permission was obtained from the School of Health Sciences, Wuhan University. Patients with hypertension and who agreed to participate in the study were included and patients with major co-morbidity such as a major psychiatric problem or a cardiac problem were excluded. Written informed consent was taken from each participant.

Data collection was done by filling the questionnaire and it was pretested among 10 hypertensive patients. The questionnaire was further evaluated by the research expert and supervisor. The final questionnaire consists of two parts. In the first part, the socio-demographic questionnaire consisted of 16 questions about the socio-demographic characteristics of the respondents. The second part consisted of five questions about the knowledge related to diet. Each correct answer for knowledge was given a score of 1 and the wrong answer was given a score of 0. Knowledge scores were divided into good scorer who scored more than 3 marks and poor scorer who scored up to 3 marks.^4^

Convenient sampling was done and the sample size was calculated using a formula,

n = Z^2^ × p × q / e^2^

   = (1.96)^2^ × 0.5 × 0.5/(0.08)^2^

   = 150.06

   = 150

where,

n = desired sample size
Z= 1.96 at 95% confidence interval p= proportion of people having good knowledge (50%)
q= 1-p
e = margin of error
Non- response rate was adjusted at 12% of the sample size, the calculated sample size was 169.

Selection and information bias has been minimized as possible. Data entry was done in Statistical Package for the Social Sciences (SPSS version 16.0). Point estimate at 95% CI was calculated along with frequency and proportion for binary data and analysis was done.

## RESULTS

Out of total 169 hypertensive patients enrolled in this study, only 79 (46.7%) had good knowledge and 90 (53.3%) had poor knowledge regarding the diet. The mean age of participants was 54.68±13.91 years. There were 58 (34.3%) males and 111 (65.7%) females. Most of the respondents 166 (98.2%) were married. The number of respondents who were illiterate was 68 (40.2%). In terms of ethnic groups, 70 (41.4%) respondents were Janajati. A majority of the respondents 145 (85.8%) belong to the Hindu followed by Buddhism 15 (8.9%) (Table 1).

**Table 1:** Socio-demographic characteristics of the participants (n=169).

Variables	n (%)
Age	
20-29	4 (2.4)
30-39	19 (11.2)
40-49	43 (25.4)
50 and above	103 (60.9)
Mean age	54.68±13.91
Gender	
Male	58 (34.3)
Female	111 (65.7)
Marital status	
Married	166 (98.2)
Unmarried	3 (1.8)
Education status	
Literate	101 (59.8)
Illiterate	68 (40.2)
Ethnicity	
Janajati	70 (41.4)
Chhetri	65 (38.5)
Brahmin	27 (16.0)
Dalit	1 (0.6)
Madhesi	1 (0.6)
Others	5 (3.0)
Religion	
Hinduism	145 (85.8)
Buddhism	15 (8.9)
Christianity	5 (3.0)
Others	4 (2.4)
Smoking	
Never	120 (71.0)
Past smoker	35 (20.7)
Present smoker	14 (8.3)

**Figure 1:**
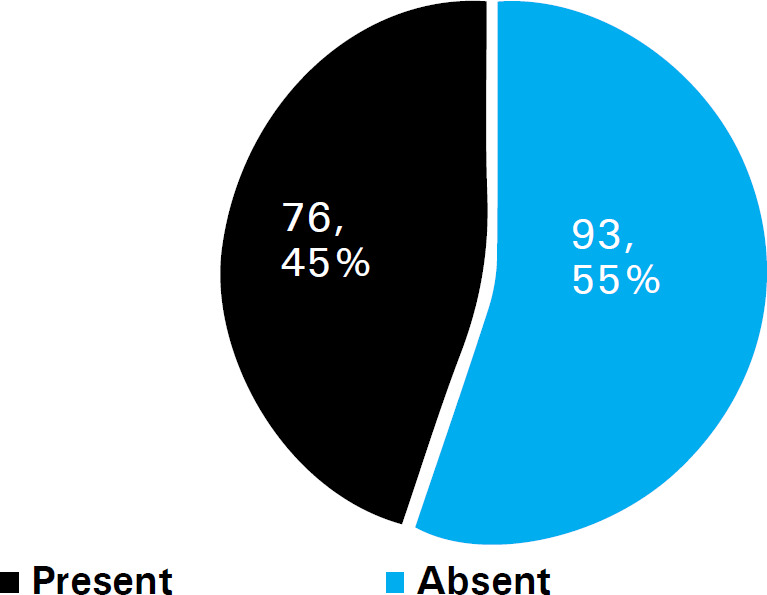
Family history of hypertension.

**Figure 2:**
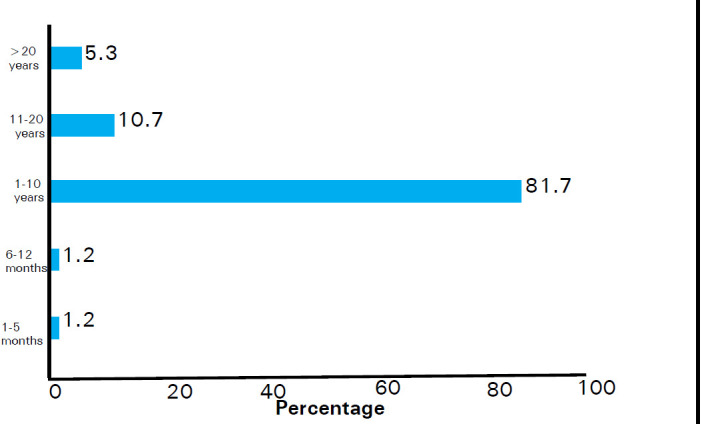
Duration of hypertension.

Most of the respondents 93 (55.0%) had a family history of hypertension (Figure 1) and the majority of the respondents 138 (81.7%) have had hypertension for about 1-10 years (Figure 2).

Out of total participants, only 79 (46.7%) had good knowledge and 90 (53.3%) had poor knowledge (Table 2).

**Table 2:** Knowledge level of participants.

Knowledge score	n (%)
Good scorer	79 (46.7)
Poor scorer	90 (53.3)

## DISCUSSION

Out of total 169 patients enrolled in this study, only 79 (46.7%) had good knowledge and 90 (53.3%) had poor knowledge regarding the diet. This is similar to a study conducted in India which also found a poor score of KAP among hypertensive patients.^5^ The majority of the participants were female in our study. The prevalence of hypertension among females was found to be higher than males in our study. Similar results were reported in studies in Srilanka^6^, Nigeria^7^, India^8^, and Botswana.^9^ The current study results revealed that the majority of the participants were aged 50 and above years which is in line with a study conducted in Egypt.^10^ A study carried out in the USA to determine the prevalence of hypertension by age and gender, they observed a high prevalence of hypertension in older adults.^11^

Smoking and alcohol consumption is considered a risk factor for hypertension. In our study, 120 (71.0%) were never smokers, 35 (20.7%) were past smokers and 14 (8.3%) were present smokers. The present smoking rate in our study is lower than the national survey in 2013 which was 18.5%^12^ and the study conducted on prevalence and awareness of hypertension among adults in Nepal which showed 23.5%.^13^ The study conducted by Shrestha S, et al. also illustrated the association of hypertension with smoking and alcohol consumption. Although the participants in our study knew about the symptoms of hypertension, smoking and alcohol consumption are correlated with hypertension but most of the participants showed a lack of hypertension complications. Only about 13.0% of participants in our study know about the complications of hypertension.

Previous research showed that lifestyle modification plays an important role in the control of hypertension CVDs.^14^ Regarding diet and exercise, about 88.2% of respondents in our study had reported avoiding extra added salty diet and 88.8% avoiding oily foods. This was similar to a study done in China that found that 81.1% of hypertensives avoiding extra salt during cooking and eating.^15^ Another study conducted in India also revealed that 75% of hypertensives in their study were avoiding extra salt in their diet.^16^ Only about 58.0% of respondents in this study doing exercise regularly. The reasons for not exercising: lack of time due to their household works, pain in legs and due to old age. Our finding is lower than the previous study conducted in India which found that about 89% of participants were physically active. A study conducted on knowledge and lifestyle factors of hypertensive subjects demonstrated that about 63% of participants were regular exercisers.^17^ If the patient is provided with proper knowledge regarding drug therapy and lifestyle modification, hypertension can be control. Therefore, there is a need for education and awareness programs for the management of hypertension.

The current study was conducted only in one center and hence the study findings cannot be generalized.

## CONCLUSIONS

This study revealed that the knowledge about diet among hypertensive patients is poor and highlighted the need for an awareness program and educational intervention for the prevention and management of hypertension. The patients should be counseled during every visit in order to obtain better outcomes and should encourage them to change in behavior and to adopt healthy practices.
